# The *C. elegans* cGMP-Dependent Protein Kinase EGL-4 Regulates Nociceptive Behavioral Sensitivity

**DOI:** 10.1371/journal.pgen.1003619

**Published:** 2013-07-11

**Authors:** Michelle C. Krzyzanowski, Chantal Brueggemann, Meredith J. Ezak, Jordan F. Wood, Kerry L. Michaels, Christopher A. Jackson, Bi-Tzen Juang, Kimberly D. Collins, Michael C. Yu, Noelle D. L'Etoile, Denise M. Ferkey

**Affiliations:** 1 Department of Biological Sciences, State University of New York at Buffalo, Buffalo, New York, United States of America; 2 Department of Cell and Tissue Biology, University of California, San Francisco, California, United States of America; Stanford University School of Medicine, United States of America

## Abstract

Signaling levels within sensory neurons must be tightly regulated to allow cells to integrate information from multiple signaling inputs and to respond to new stimuli. Herein we report a new role for the cGMP-dependent protein kinase EGL-4 in the negative regulation of G protein-coupled nociceptive chemosensory signaling. *C. elegans* lacking EGL-4 function are hypersensitive in their behavioral response to low concentrations of the bitter tastant quinine and exhibit an elevated calcium flux in the ASH sensory neurons in response to quinine. We provide the first direct evidence for cGMP/PKG function in ASH and propose that ODR-1, GCY-27, GCY-33 and GCY-34 act in a non-cell-autonomous manner to provide cGMP for EGL-4 function in ASH. Our data suggest that activated EGL-4 dampens quinine sensitivity via phosphorylation and activation of the regulator of G protein signaling (RGS) proteins RGS-2 and RGS-3, which in turn downregulate Gα signaling and behavioral sensitivity.

## Author Summary

All animals rely on their ability to sense and respond to their constantly changing environments to survive. *C. elegans* (small roundworms) depend heavily upon their ability to taste and smell chemical information in their soil environment to find food and avoid danger. While similar signal transduction pathways are implicated in both *C. elegans* and vertebrate chemosensation, there are still large gaps in our understanding of the mechanisms used to regulate signaling in these systems. We have identified a new role for the *C. elegans* cGMP-dependent protein kinase (PKG) EGL-4 in the negative regulation of nociceptive chemosensory signaling. Our data suggest that EGL-4 negatively regulates signaling and behavior by activating known inhibitors of G protein-coupled signal transduction, RGS proteins. Using *C. elegans* behavioral response to aversive stimuli as the readout for neuronal activity, we provide the first evidence for PKG regulation of RGS function in sensory neurons in any system.

## Introduction

The ability to detect and avoid noxious stimuli in the environment is critical to an organism's survival. Nociceptive sensory systems mediate detection of harmful stimuli, allowing rapid initiation of protective behavioral responses. In the nematode *Caenorhabditis elegans*, the pair of polymodal nociceptive head sensory neurons termed ASH respond to a broad range of aversive stimuli, including soluble chemicals (e.g. the bitter tastant quinine, heavy metals and SDS), odorants (e.g. octanol), ions (e.g. Na^+^), osmotic stress and mechanosensory stimulation (nose touch) [1]–[9]. Because the ASH sensory neurons synapse onto command interneurons that drive backward locomotion via their connections with motor neurons, ASH activation elicits reversal and stimulus avoidance.

In general, olfaction and some forms of taste (including bitter) are mediated by G protein-coupled signal transduction pathways [10]–[12]. Signaling is initiated when the chemosensory ligand binds to a seven-transmembrane G protein-coupled receptor (GPCR). This induces a conformational change in the receptor that activates the associated heterotrimeric G proteins. Gα becomes active upon exchange of GDP for GTP. Once dissociated, the Gα-GTP and Gβγ subunits can activate distinct signaling cascades within the cell [13]. Although the *C. elegans* genome encodes >500 predicted functional chemosensory GPCRs [14], only one aversive chemical stimulus, dihydrocaffeic acid, has been functionally coupled to a receptor, DCAR-1 [15]. However, the *C. elegans* stimulatory Gα subunits ODR-3 and GPA-3 (both most similar to Gα_i/o_) are used by ASH to mediate avoidance of a variety of stimuli [7], [8], [16]–[18].

Regulator of G protein signaling (RGS) proteins are important negative regulators of G protein-coupled signal transduction. They bind to Gα-GTP and accelerate the intrinsic GTPase activity of the Gα subunits. Once GTP is hydrolyzed (back to GDP), signaling via Gα is attenuated [19], [20]. By dampening Gα signaling, RGS proteins help to protect cells from overstimulation. Mammalian RGS proteins have been implicated in the regulation of sensory signaling. For example, RGS9-1 plays an important role in regulating the light response of rod photoreceptor cells. Retinas isolated from knock-out mice lacking RGS9-1 function displayed a prolonged dim flash response [21], while overexpression of the RGS9-1 containing complex resulted in a faster light response recovery in the retina rod cells of transgenic mice [22]. In addition, RGS21 is coexpressed with T2R bitter receptors and T1R2 and T1R3 sweet receptors in rat taste bud cells [23]. RGS21 also coprecipitates with α-gustducin, the Gα_i_ protein that is coupled to T2R bitter receptors [23]–[27]. Taken together, these observations suggest a potential role for RGS21 in the regulation of taste transduction.

*C. elegans* lacking RGS-3 function are defective in their response to a subset of strong sensory stimuli detected by the ASH sensory neurons [28]. Interestingly, the behavioral defects appear to be due to increased signaling in the sensory neurons that in turns leads to decreased synaptic transmission [28]. Although our previous study did not identify chemosensory hypersensitivity (e.g. enhanced sensitivity to dilute quinine) in *rgs-3* mutant animals, we note that feeding status and, consequently, biogenic amine (e.g. dopamine and serotonin) levels modulate *rgs-3* behavioral responses [28]. For example, *rgs-3* animals responded to 100% octanol (odorant) and 10 mM quinine (tastant) when they were assayed in the presence of food (*E. coli* bacterial lawn), and were only defective when assayed after a short (10 minute) period of starvation [28]. Taken together, the sensitivity of *C. elegans* to an environmental stimulus is ultimately coordinated by proteins (e.g. RGS-3) that directly regulate the sensory G protein-coupled signaling cascade, in conjunction with signals modulated by nutritional status. All of the tastant avoidance experiments presented herein were performed 30 minutes after animals were removed from food, in contrast to the previous study focused on *rgs-3*, where “off food” assays were performed 10 minutes after animals were removed from the bacterial food source [28].

cGMP-dependent protein kinases (PKGs) are serine/threonine kinases that are activated upon the binding of cGMP [29], [30] and their function is implicated in a variety of cellular contexts. PKGI-null mice have altered physiological processes including impaired vasorelaxation [31]–[33] and increased platelet aggregation and activation [34] via the nitric oxide/cGMP/PKGI signaling pathway [35]. They also display impaired nociceptive flexion reflexes [36] and reduced inflammatory hyperalgesia [37]. Mice lacking PKGII function exhibit enhanced anxiety and alcohol consumption [38]. In addition, mammalian PKG can act as an indirect negative regulator of G protein-coupled signal transduction. Upon activation, PKGI phosphorylates serine/threonine residues on RGS2 in mouse vascular smooth muscle cells [39] and Rat1 fibroblast cells [40], RGS3 and RGS4 in rat diencephalic astrocytes [41] and RGS4 in rabbit gastric smooth muscle cells [42]. Phosphorylation by PKG causes each RGS protein to translocate from the cytosol to the plasma membrane, stimulating its binding to Gα_q_ and consequently enhancing GTPase activity and the dampening of Gα signaling [39]–[42].

In *C. elegans*, EGL-4 is a cGMP-dependent protein kinase with physiological roles including egg laying, life span, cell growth, quiescence and dauer formation [43]–[46]. Additionally, EGL-4 function contributes to *C. elegans* sensory responses. For example, *egl-4(lof)* animals are strongly defective for chemotaxis to the AWA-detected odorant diacetyl [47]. In the AWC olfactory neurons, EGL-4 functions in the cytoplasm and nucleus to regulate short term and long term olfactory adaptation, respectively [48], [49]. EGL-4 also acts with KIN-29, a salt-inducible kinase, to regulate chemoreceptor gene expression [50], [51], contributing to behavioral plasticity and chemosensory signal-dependent development [52].

In mammalian systems, cGMP binding activates PKG [53]–[55] by relieving an inhibitory interaction that blocks the kinase domain [56]. *C. elegans* EGL-4 has two allosteric cGMP-binding domains in its amino-terminal half [46] and altering key residues within either or both cGMP-binding domains renders animals completely defective for AWC-mediated odor adaptation [49]. Interestingly, while EGL-4 requires intact cGMP binding domains to accumulate in the AWC nucleus [49], it requires reduction in cGMP levels to enter the AWC nucleus and promote adaptation [57]. In uterine epithelial cells, nuclear EGL-4 regulates gene expression [58]. Guanylyl cyclases (GCYs) produce cGMP, and 27 receptor-type GCYs and 7 soluble GCYs are encoded by the *C. elegans* genome [59]. Although several GCYs have been shown to function in *C. elegans* sensory neurons, no GCYs are known to be expressed in or function in ASH.

Herein we describe a new role for the *C. elegans* cGMP-dependent protein kinase EGL-4 as a negative regulator of nociceptive chemosensory signaling. Animals lacking EGL-4 function respond better than wild-type animals to dilute concentrations of several ASH-detected chemical stimuli, including the bitter tastant quinine. We provide evidence that a subset of GCYs function non-cell-autonomously to generate cGMP to stimulate EGL-4 phosphorylation and activation of RGS proteins in ASH to dampen G protein-coupled chemosensory signal transduction and behavioral sensitivity.

## Results

### EGL-4 Regulates Behavioral Sensitivity to Quinine

**Figure 1:**
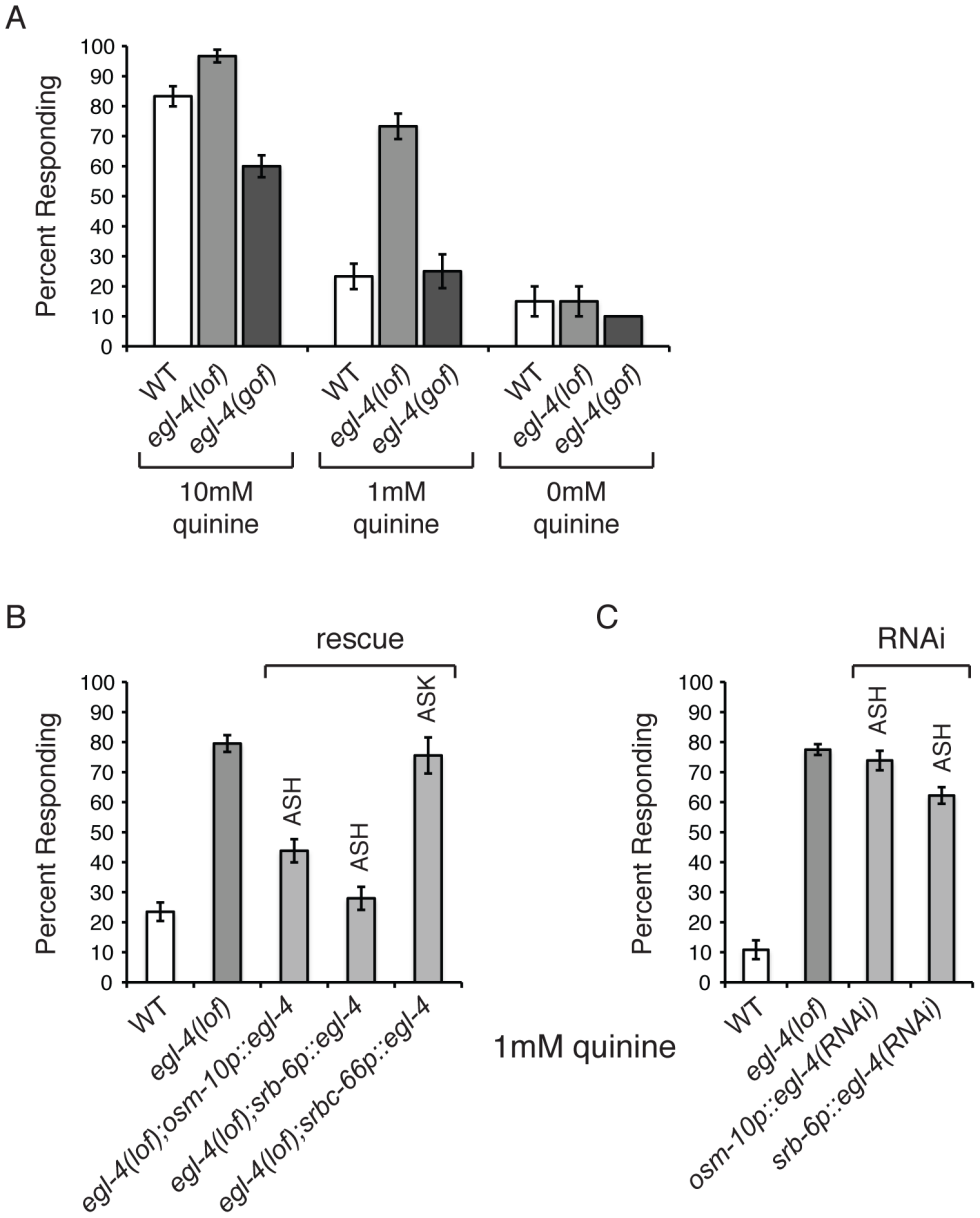
*C. elegans* EGL-4 regulates quinine sensitivity in ASH. (A) *egl-4(lof)* animals respond better than wild-type animals to dilute (1 mM) quinine, while *egl-4(gof)* animals show a decreased sensitivity to 10 mM quinine, when compared to wild-type animals. p<0.0001 for each. (B) The ASH sensory neurons are the primary neurons used to detect quinine, but the ASK neurons also contribute [7]. The *osm-10* [3], *srb-6* [61] and *srbc-66* [62] promoters were used to drive expression of wild-type *egl-4* in *egl-4(lof)* animals. The *osm-10* promoter expresses in ASH, ASI, PHA and PHB, while the *srb-6* promoter drives expression in ASH, ADL, ADF, PHA and PHB. ASH is the only head sensory neuron common to both promoters. The *srbc-66* promoter expresses in ASK. While *egl-4(lof)* animals respond better than wild-type animals to 1 mM quinine, restoring EGL-4 function in ASH significantly diminished this hypersensitivity (p<0.0001 for both). EGL-4 expression in ASK had no effect (p>0.5). (C) RNAi knock-down of *egl-4* in the ASH sensory neurons of otherwise wild-type animals, using the *osm-10* [3] or *srb-6* [61] promoter resulted in behavioral hypersensitivity to dilute (1 mM) quinine, similar to *egl-4(lof)* animals (p<0.0001 when compared to N2 animals for both transgenes). The percentage of animals responding is shown. The combined data of ≥3 independent lines, n≥120 transgenic animals, is shown. Error bars represent the standard error of the mean (SEM). Alleles used: *egl-4(n479)* loss-of-function and *egl-4(ad450)* gain-of-function. WT = the N2 wild-type strain. lof = loss-of-function. gof = gain-of-function.

EGL-4 has not previously been implicated in nociceptive chemosensory signaling in *C. elegans* and *egl-4(n479)* loss-of-function (lof) animals effectively avoid the bitter tastant 10 mM quinine (Figure 1A). However, we noticed that *egl-4(lof)* animals appeared to respond to 10 mM quinine slightly better than wild-type animals (p<0.01). To determine whether EGL-4 might contribute to chemosensory signaling in a way that might not be fully revealed by this high concentration of quinine, which elicits a robust behavioral response even in wild-type animals, we challenged both genotypes with dilute (1 mM) quinine. At this lower concentration, *egl-4(lof)* animals were clearly hypersensitive, with significantly more *egl-4(lof)* animals responding to the dilute quinine than wild-type animals (Figure 1A). As loss of EGL-4 function led to quinine hypersensitivity, we reasoned that excess EGL-4 function could result in diminished quinine sensitivity. *ad450* is a gain-of-function (gof) allele of *egl-4*. Consistent with our prediction, *egl-4(gof)* animals were no longer hypersensitive to quinine and responded worse than wild-type animals to 10 mM quinine (Figure 1A).

The ASH sensory neurons are the main cells used to detect quinine in *C. elegans*, but the ASK neurons also contribute [7]. EGL-4 is broadly expressed throughout the animal, including these sensory neurons [60]. To determine whether EGL-4 function in either the ASHs or ASKs is sufficient to regulate quinine response, the cell-selective promoters *osm-10* (ASH) [3], *srb-6* (ASH) [61] and *srbc-66* (ASK) [62] were used to restore EGL-4 function in each neuron pair and animals were assayed for response to 1 mM quinine. Expression in ASH, but not ASK, returned the *egl-4(lof)* response to wild-type levels so that animals were no longer hypersensitive to the dilute stimulus (Figure 1B). To assess whether selective loss of EGL-4 function in the ASH sensory neurons could also lead to quinine hypersensitivity, we used the cell-specific RNAi approach of Esposito et al. [63]. The *osm-10* [3] and *srb-6* [61] promoters were used to co-express a non-coding fragment of *egl-4* in both the sense and antisense orientations in the ASH neurons of otherwise wild-type animals. *egl-4* knock-down using either promoter resulted in hypersensitivity to 1 mM quinine, similar to *egl-4(lof)* animals (Figure 1C). Taken together, our data suggest a role for EGL-4 in the negative regulation of quinine avoidance in the ASH sensory neurons.

### EGL-4 Does Not Regulate ASH Sensitivity in General

**Figure 2:**
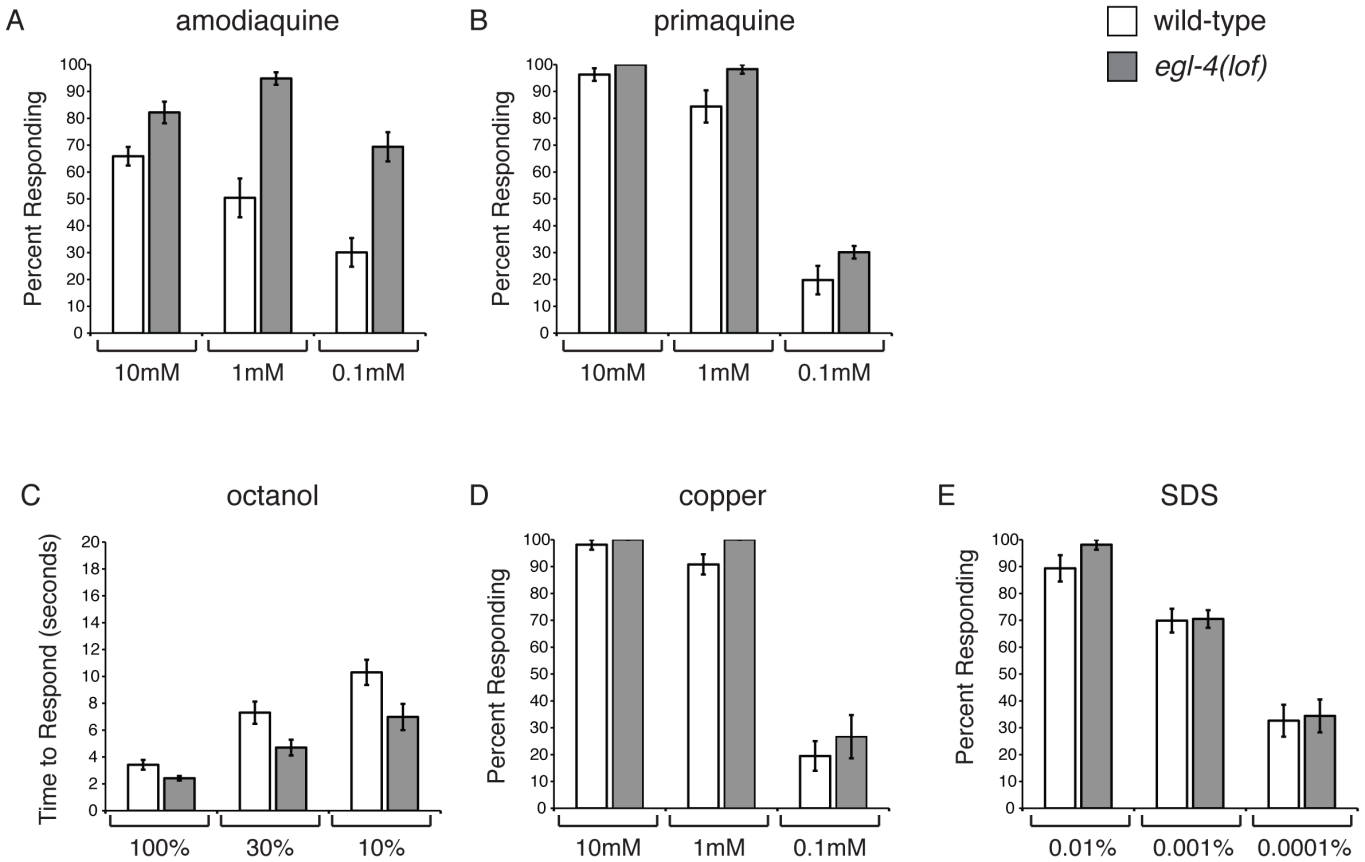
EGL-4 does not regulate ASH sensitivity in general. *C. elegans* respond to bitter stimuli in addition to quinine. (A) Animals lacking EGL-4 function are hypersensitive to dilute amodiaquine (p≤0.01 when compared to wild-type animals), but not dilute primaquine (B) (p≥0.05). The percentage of animals responding is shown. The ASH sensory neurons also detect the volatile odorant octanol, the heavy metal copper and the detergent SDS. (C) *egl-4(lof)* mutant animals are moderately hypersensitive to dilute octanol (p<0.02). Time to respond is shown. (D–E) *egl-4(lof)* animals respond similarly to wild-type animals to both copper and SDS, across a range of concentrations (p>0.1 for each concentration, except p = 0.03 for 1 mM copper). The percentage of animals responding is shown. n>40 for each. All tastants were dissolved in M13 buffer, pH 7.4. Error bars represent the standard error of the mean (SEM). Allele used: *egl-4(n479)* loss-of-function. WT = the N2 wild-type strain. lof = loss-of-function.

In addition to the ASH-mediated avoidance of quinine, *C. elegans* avoid several additional bitter compounds [7]. We therefore tested whether EGL-4 selectively regulates quinine avoidance (Figure 1) or whether it could regulate bitter taste responses generally. We assayed the response of *egl-4(lof)* animals to the bitter tastants amodiaquine and primaquine (Figures 2A and 2B). *egl-4(lof)* animals were hypersensitive to amodiaquine, with more animals responding than wild-type across a range of concentrations (Figure 2A). However, *egl-4(lof)* animals responded to primaquine similarly to wild-type animals and did not appear hypersensitive (Figure 2B).

In *C. elegans*, as in mammals, bitter compounds signal through G protein-coupled receptor pathways [7], [10], [11]. The aversive odorant 1-octanol, which like quinine is detected primarily by the ASH sensory neurons [61], [64], also activates G protein-coupled signaling [16], [65]. To determine whether EGL-4 regulates octanol avoidance, animals were assayed for their time to reverse when presented with 100%, 30% or 10% octanol. At each concentration *egl-4(lof)* animals responded better than wild-type animals (Figure 2C), suggesting that EGL-4 normally dampens this olfactory response.

The ASH sensory neurons also detect soluble stimuli (copper and SDS) that are thought to signal in a G protein-independent manner [4], [6], [8]. To assess whether EGL-4 regulation of sensory signaling extends to these compounds, animals were tested for their avoidance response across a range of concentrations for each. In all cases, the response of *egl-4(lof)* animals was similar to that of wild-type animals (Figures 2D and 2E). We conclude that EGL-4 regulates response to a subset of G protein-coupled chemosensory responses, including the bitter tastants quinine and amodiaquine and the aversive odorant octanol, but does not regulate ASH sensitivity in general.

### EGL-4 Functions in the Cytoplasm of the ASH Sensory Neurons to Regulate Quinine Sensitivity and Calcium Signaling

**Figure 3:**
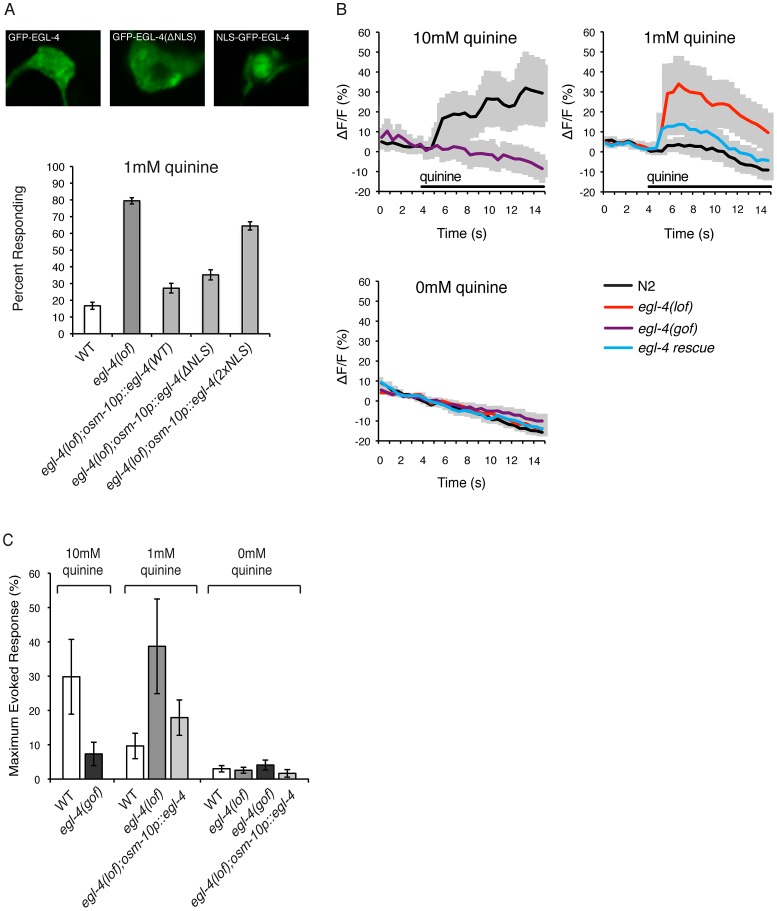
EGL-4 functions in the cytoplasm to regulate calcium signaling. (A) EGL-4 functions in the cytoplasm to regulate behavioral sensitivity to quinine. The *osm-10* promoter [3] was used to express wild-type EGL-4, EGL-4 lacking its endogenous nuclear localization sequence (NLS) or EGL-4 with an additional NLS in the ASH sensory neurons of *egl-4(lof)* animals. In each case, the EGL-4 was expressed as a fusion with the green fluorescent protein (GFP). Wild-type GFP−EGL-4 was localized throughout the cell and rescued the quinine hypersensitivity of *egl-4(lof)* animals. GFP−EGL-4(ΔNLS) was restricted to the cytoplasm and rescued the hypersensitivity of *egl-4(lof)* animals as well as wild-type GFP−EGL-4 (p>0.05). NLS−GFP−EGL-4 was sequestered to the nucleus and had only a small, but statistically significant, rescuing effect (p<0.001). (B) Stimulus-evoked calcium transients in the ASH neurons are enhanced in *egl-4(lof)* animals. The *sra-6* promoter [61] was used to express the genetically encoded calcium indicator G-CaMP3 [69] in the ASH sensory neurons, *kyEx2865* (*sra-6p::G-CaMP3;ofm-1p::gfp*) [70]. Using a microfluidic device, an adult animal was restrained while quinine was delivered to its nose for 10 seconds (black horizontal bar), and the change in fluorescence intensity was recorded. The average ratio change ± the standard error of the mean (SEM) is indicated on each trace. n = 10 animals for each condition. (C) The averaged maximum evoked calcium change for 10, 1 and 0 mM quinine is shown. *egl-4(lof)* animals showed an elevated ASH calcium flux upon exposure to 1 mM quinine when compared to wild-type animals (p<0.05) that is rescued by *osm-10p::egl-4* expression in ASH (p>0.1 when compared to wild-type animals). Error bars represent the SEM. Alleles used: *egl-4(n479)* loss-of-function and *egl-4(ad450)* gain-of-function. WT = the N2 wild-type strain. lof = loss-of-function, gof = gain-of-function. s = seconds.

Upon prolonged exposure to attractive odorants, EGL-4 translocates from the cytoplasm into the nucleus of the AWC olfactory neurons to regulate long-term adaptation in a manner downstream of primary sensory transduction [49]. To determine where within the ASH sensory neurons EGL-4 might function to regulate quinine sensitivity, we used the *osm-10* promoter [3] to express a functional GFP−EGL-4 fusion protein [49] and visualized its subcellular localization via GFP fluorescence. As was previously reported for naïve AWC neurons [49], [66], GFP−EGL-4 was distributed throughout ASH in wild-type animals, with expression seen in both the cytoplasm and the nucleus (Figure 3A).

To ascertain whether the cytoplasmic or nuclear pools of EGL-4 contribute to the regulation of quinine avoidance, we used the *osm-10* promoter [3] to express two modified forms of GFP−EGL-4 in *egl-4(lof)* animals. Deletion of the endogenous nuclear localization sequence, GFP−EGL-4(ΔNLS), lead to cytoplasmic accumulation and had no effect on the ability of EGL-4 to rescue the quinine hypersensitivity of *egl-4(lof)* animals (Figure 3A). Conversely, *egl-4(lof)* animals expressing the constitutively nuclear NLS−GFP−EGL-4 remained hypersensitive in their response to 1 mM quinine (Figure 3A). Combined, these results suggest a primarily cytoplasmic role for EGL-4 in the negative regulation of quinine signaling.

Genetically encoded calcium indicators such as G-CaMP [67] and cameleon [68] can be used to monitor the activity of *C. elegans* neurons. In response to quinine, a transient rise in intracellular calcium levels has been seen in the ASHs of wild-type animals [8], [17], [65]. We imaged quinine-evoked calcium fluxes in wild-type, *egl-4(lof)* and *egl-4(gof)* animals expressing G-CaMP3 in ASH (*sra-6p::G-CaMP3*) [69], [70]. Consistent with their observed behavioral hypersensitivity (Figures 1 and 3A), *egl-4(lof)* animals displayed quinine-evoked ASH calcium fluxes of greater amplitude in response to 1 mM quinine, while expression of wild-type *egl-4* in ASH, using the *osm-10* promoter [3], rescued this elevated calcium flux (Figures 3B and 3C). Conversely, *egl-4(gof)* animals showed a diminished calcium flux in response to 10 mM quinine (Figures 3B and 3C), consistent with their decreased behavioral sensitivity to this concentration (Figure 1). Taken together, we suggest that the elevated calcium fluxes seen in the absence of EGL-4 function underlie the enhanced sensitivity to quinine.

### RGS Proteins Are Targets of EGL-4

**Figure 4:**
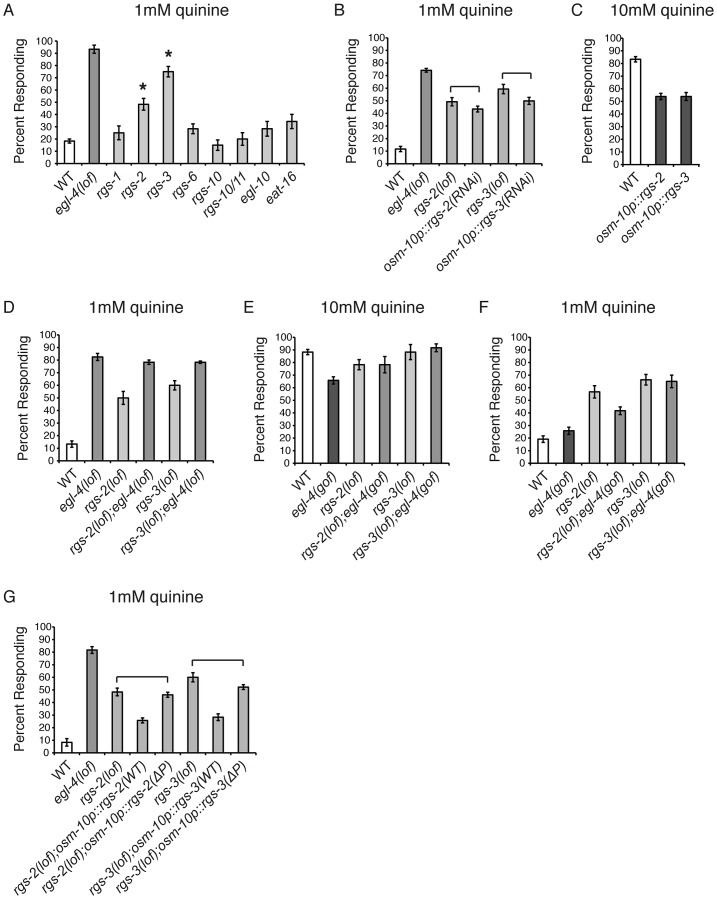
RGS proteins are targets of EGL-4. (A) Animals lacking each of the 8 neuronally expressed RGS proteins were tested for response to 1 mM quinine. *rgs-2(lof)* and *rgs-3(lof)* animals respond better than wild-type animals to dilute (1 mM) quinine (p<0.001). (B) RNAi knock-down of *rgs-2* or *rgs-3* in the quinine-detecting ASH sensory neurons of otherwise wild-type animals, using the *osm-10* promoter [3], resulted in behavioral hypersensitivity to dilute (1 mM) quinine, similar to *rgs-2(lof)* and *rgs-3(lof)* animals, respectively (p>0.05 for both transgenes when compared to the respective *rgs* loss-of-function animals). The *srb-6* promoter [61] was also used for ASH knock-down of *rgs-3*, and similarly resulted in hypersensitivity to 1 mM quinine (data not shown). (C) Wild-type animals overexpressing ectopic *rgs-2* or *rgs-3* cDNA displayed diminished response to 10 mM quinine (p<0.0001 when compared to wild-type animals). (D) *rgs-2(lof);egl-4(lof)* and *rgs-3(lof);egl-4(lof)* double mutant animals responded to dilute (1 mM) quinine similarly to *egl-4(lof)* animals (p>0.1 for each). (E) *egl-4(gof)* animals lacking either RGS-2 or RGS-3 function responded to 10 mM quinine similarly to the *rgs-2(lof)* and *rgs-3(lof)* animals, respectively (p>0.5) and (F) were hypersensitive to 1 mM quinine (p<0.001 when compared to wild-type animals). (G) *rgs-2(lof)* and *rgs-3(lof)* animals are hypersensitive to dilute (1 mM) quinine. ASH expression of wild-type RGS-2 or RGS-3, using the *osm-10* promoter [3], rescued quinine hypersensitivity in the respective loss-of-function animals. The predicted PKG phosphorylation target site in each was mutated (ΔP), making RGS-2(S126A) and RGS-3(S154A). *rgs-2(lof)* animals expressing RGS-2(ΔP) and *rgs-3(lof)* animals expressing RGS-3(ΔP) remained hypersensitive to dilute quinine (p>0.05 for each). The percentage of animals responding is shown. The combined data of ≥3 independent lines, n≥120 transgenic animals, is shown. Error bars represent the standard error of the mean (SEM). Alleles used: *egl-4(n479)*, *rgs-1(nr2017)*, *rgs-2(vs17)*, *rgs-3(vs19)*, *rgs-6(vs62)*, *rgs-10(ok1039)*, *rgs-10/11(vs109)*, *egl-10(md176)* and *eat-16(tm761)* loss-of-function and *egl-4(ad450)* gain-of-function. WT = the N2 wild-type strain. lof = loss-of-function. gof = gain-of-function.

PKGs have been shown to down-regulate Gα_q_ signaling by directly phosphorylating and activating RGS proteins in mouse vascular smooth muscle cells, Rat1 fibroblast cells, rat diencephalic astrocytes, and rabbit gastric smooth muscle cells [39]–[42]. The *C. elegans* genome encodes twelve predicted RGS proteins [71]. To determine if the loss of RGS function also results in quinine hypersensitivity, similar to loss of EGL-4, we tested animals lacking each of the eight neuronally expressed RGS proteins for response to 1 mM quinine (Figure 4A). *rgs-2(lof)* and *rgs-3(lof)* animals displayed a statistically significant hypersensitive response, with more animals than wild-type responding to the dilute stimulus. Although an RGS-2 reporter construct did not show expression in ASH [72], RGS-3 is known to be expressed in and function in the quinine-detecting ASH sensory neurons [28]. We used the *osm-10* promoter [3] to conduct cell-selective knock-down of *rgs-2* and *rgs-3* in the ASH sensory neurons of wild-type animals and in both cases, knock-down also resulted in hypersensitivity to dilute quinine (Figure 4B). Furthermore, consistent with a role in dampening sensory sensitivity, overexpression of RGS-2 or RGS-3 in the ASHs of wild-type animals decreased quinine response (Figure 4C).

If EGL-4 functions in the same pathway as RGS-2 and RGS-3 to regulate chemosensory signaling, then *egl-4(lof);rgs-2(lof)* and *egl-4(lof);rgs-3(lof)* double mutant animals should display a behavioral phenotype similar to *egl-4(lof)* animals and the hypersensitivity should not be additive. We found that the double mutant animals' response to dilute (1 mM) quinine was indistinguishable from animals lacking only EGL-4 function (Figure 4D), suggesting that they do function in the same regulatory pathway. To determine whether EGL-4 might function upstream of the RGS proteins, we utilized the *egl-4(gof)* animals, which display reduced sensitivity to quinine (Figure 1A). If RGS-2 and RGS-3 function downstream of EGL-4, then loss of either in combination with the *egl-4(gof)* allele should relieve the dampened sensitivity of *egl-4(gof)* mutants in response to 10 mM quinine. Indeed, *egl-4(gof);rgs-2(lof)* and *egl-4(gof);rgs-3(lof)* double mutants both responded to 10 mM quinine similarly to the *rgs* single mutants (Figure 4E). In addition, the double-mutant animals were hypersensitive to 1 mM quinine, similar to the *rgs* single mutants (Figure 4F).

The characterized consensus sequence of mammalian PKG, seen in 75% of targets surveyed, consists of (R/K)_2–3_-X-S*/T* [73]–[76]. A search for putative PKG phosphorylation sites in RGS-2 and RGS-3 using NetPhosK 1.0 server [77] revealed one site in each protein, serine 126 in RGS2 and serine 154 in RGS-3. Both of the predicted target serines were changed to alanine and the mutated constructs, expressed under the control of the *osm-10* promoter [3], were compared to the wild-type cDNAs for their ability to rescue quinine hypersensitivity. If EGL-4 phosphorylates RGS-2 and RGS-3 to stimulate their activity, loss of the target phosphorylation site(s) in each RGS protein should preclude activation. This would result in an RGS protein that cannot rescue the hypersensitivity of the animals lacking the corresponding RGS protein. As shown in Figure 4G, ASH expression of wild-type RGS-2 or RGS-3 in the respective loss-of-function animals, using the *osm-10* promoter [3], returned quinine sensitivity to the levels of wild-type animals. However, *rgs-2(lof)* animals expressing RGS-2(S126A) and *rgs-3(lof)* animals expressing RGS-3(S154A) remained hypersensitive to quinine, suggesting that these putative PKG phosphorylation sites are required for RGS-2 and RGS-3 function. We conclude that EGL-4 functions upstream of RGS-2 and RGS-3, and propose that phosphorylation by EGL-4 may be required for the function of these regulatory proteins in ASH.

### The Guanylyl Cyclases ODR-1, GCY-27, GCY-33 and GCY-34 Act Upstream of EGL-4

**Figure 5:**
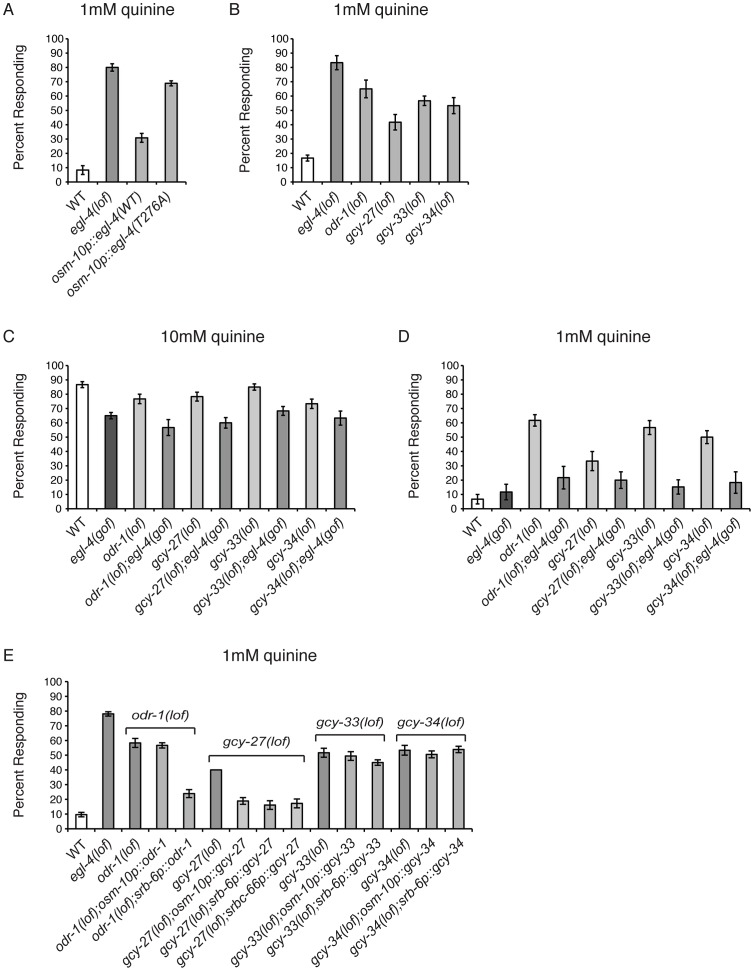
Guanylyl cyclases act upstream of EGL-4. (A) The *osm-10* promoter [3] was used to express wild-type EGL-4 or the cGMP binding mutant EGL-4(T276A) in the ASH sensory neurons of *egl-4(lof)* animals. While wild-type EGL-4 significantly rescued the *egl-4(lof)* quinine hypersensitivity (p<0.001), *egl-4(lof)* animals expressing EGL-4(T276A) remained hypersensitive. (B) Loss-of-function mutations in the guanylyl cyclase genes *odr-1*, *gcy-27*, *gcy-33* and *gcy-34* resulted in behavioral hypersensitivity to dilute (1 mM) quinine (p<0.01 for each). *egl-4(gof)* animals lacking ODR-1, GCY-27, GCY-33 or GCY-34 function showed diminished sensitivity to both (C) 10 mM quinine and (D) 1 mM quinine, similar to *egl-4(gof)* single mutant animals (p>0.1 for each double mutant when compared to *egl-4(gof)* animals). The percentage of animals responding is shown. (E) The *osm-10* promoter [3] (ASH, ASI, PHA and PHB), *srb-6* promoter [62] (ASH, ADL, ADF, PHA and PHB), and *srbc-66* [62] (ASK) promoters were used in cell-selective rescue experiments. The quinine hypersensitivity of *odr-1(lof)* animals was rescued by *srb-6p::odr-1* expression (p<0.001), but not *osm-10p::odr-1* expression (p>0.5). *gcy-27(lof)* hypersensitivity was rescued by all three promoters (p<0.001 for each). Neither *gcy-33(lof)* nor *gcy-34(lof)* hypersensitivity was rescued using the *osm-10* or *srb-6* promoters (p>0.05 for each). The combined data of ≥3 independent lines, n≥120 transgenic animals, is shown. Error bars represent the standard error of the mean (SEM). Alleles used: *egl-4(n479)*, *odr-1(n1936)*, *gcy-27(ok3653)*, *gcy-33(ok232)* and *gcy-34(ok2953)* loss-of-function and *egl-4(ad450)* gain-of-function. WT = the N2 wild-type strain. lof = loss-of-function. gof = gain-of-function.

A single point mutation (T276A) within the cGMP-binding domain of EGL-4 abolished its function in AWC-mediated adaptation [49]. Although no guanylyl cyclase (GCY) has been reported to be expressed in ASH, we reasoned that because EGL-4 is a PKG, cGMP binding is likely required for its function in the regulation of quinine avoidance. Although ASH expression of wild-type EGL-4 significantly rescued the *egl-4(lof)* quinine hypersensitivity, *egl-4(lof)* animals expressing EGL-4(T276A) remained hypersensitive to dilute quinine (Figure 5A). This suggests that cGMP binding is required for EGL-4 function in ASH-mediated avoidance behaviors. It also suggests that one or more GCYs may provide cGMP to regulate ASH function.

We assayed animals with loss-of-function alleles for 19 of the 34 GCYs encoded by the *C. elegans* genome [59] for response to dilute (1 mM) quinine (data not shown). Four GCY mutants, *odr-1*, *gcy-27*, *gcy-33* and *gcy-34*, responded better than wild-type animals (Figure 5B). To determine whether the guanylyl cyclases ODR-1, GCY-27, GCY-33 and GCY-34 function upstream of EGL-4, we again utilized the *egl-4(gof)* animals, which display reduced sensitivity to 10 mM quinine (Figure 1A). A loss-of-function allele of each of the 4 GCYs was combined with the *egl-4(gof)* mutation and double mutant animals were assayed for quinine avoidance. Each double mutant remained less sensitive to both 10 mM and 1 mM quinine, similar to the *egl-4(gof)* single mutant animals (Figures 5C and 5D), supporting a role for ODR-1, GCY-27, GCY-33 and GCY-34 upstream of EGL-4 in the regulation of quinine sensitivity.

GCY expression has not previously been reported in ASH, consistent with our own analysis of *odr-1*, *gcy-27*, *gcy-33* and *gcy-34* GFP reporter constructs (data not shown). However, *gcy-27* is expressed in the ASK head sensory neurons [59] that also contribute to quinine response [7]. To determine whether expression of any of these GCYs in ASH was sufficient to rescue the quinine hypersensitivity of the respective loss-of-function animals, we used the *osm-10* [3] and *srb-6* [61] promoters in cell-selective rescue experiments, as their expression overlaps in ASH. The hypersensitivity of *odr-1(lof)* animals was rescued by *srb-6p::odr-1*, but not *osm-10p::odr-1* expression, and expression using neither promoter was sufficient to rescue *gcy-33(lof)* or *gcy-34(lof)* hypersensitivity (Figure 5E). Interestingly, the hypersensitivity of *gcy-27(lof)* animals was rescued by both *osm-10p::gcy-27* and *srb-6p::gcy-27*, as well as by ASK-selective expression using the *srbc-66* promoter [62] (Figure 5E). However, as these constructs contain *gcy-27* genomic sequence, we cannot rule out the possibility that regulatory information within the introns could direct expression in addition cells beyond those predicted by the cell-selective promoters used. Taken together, and consistent with the reported expression patterns, these results suggest that the primary site of GCY function is in cells other than the ASHs, and that the cyclases may function in a non-cell-autonomous manner to provide cGMP to regulate EGL-4 function in ASH.

## Discussion

### EGL-4 Functions as a Negative Regulator of Quinine Sensitivity

PKGs regulate the physiological responses of a variety of cell types, and have a wide range of known and predicted protein targets [35], [78]. As the *C. elegans* genome encodes only two PKGs, EGL-4 (also known as PKG-1) and PKG-2, it provides an excellent model environment in which to further elucidate the unique physiological roles of PKGs in different cellular contexts. In the AWC olfactory neurons, EGL-4 has been shown to regulate both short-term and long-term adaption. At the cellular level, adaptation is thought to serve as a protective measure against prolonged stimulation, as in the case of photoreceptor signaling for long-term light adaptation [79]. In *C. elegans*, short-term olfactory adaptation (<30 minutes) diminishes odorant sensitivity for up to 60 minutes and utilizes cytoplasmic EGL-4, whereas long-term adaptation to prolonged odor exposure (>80 minutes) requires the translocation of EGL-4 to the nucleus, where it can alter gene expression and decrease sensitivity to attractive scents for the lifetime of the animal (until the animal is refed) [48], [49], [66]. The cellular localization of EGL-4 depends on the levels of cGMP, as increased cGMP resulting from the loss of phosphodiesterases blocks odor-induced nuclear accumulation of EGL-4 [57], and loss of ODR-1 function leads to constitutively nuclear EGL-4 [57]. In addition, ectopic expression of constitutively nuclear EGL-4 mimicked the adapted state and decreased sensitivity to AWC-detected attractive odors [49]. However, a majority of mammalian PKG substrates are signaling proteins, suggesting an important role for PKGs in cytoplasmic cellular responses that may be closer to the initial stimulus signaling event [35], [78].

Similar to PKGI and PKGII in mammalian systems, whose targets include components of G protein-coupled signaling [39]–[42], the c-Jun N-terminal kinases (JNK) pathway [80] and the anti-apoptotic pathway that includes Bad and Akt [81], EGL-4 appears to function in or regulate multiple signal transduction pathways in *C. elegans*. For example, EGL-4 likely functions upstream of the TGF-β pathway SMAD transcription factors DAF-3 and DAF-5, as mutations in DAF-3 and DAF-5 suppress several *egl-4* mutant phenotypes, including chemosensory, dauer formation, egg laying and body size defects [47]. In addition, EGL-4 has been implicated in *C. elegans* Notch signaling in sensory neurons, acting directly or indirectly downstream of Notch receptor activation. Animals lacking the DOS-motif Notch co-ligand OSM-11 do not effectively avoid 100% octanol, and this defect is partially suppressed by the gain-of-function *egl-4(ad450)* allele [82]. We now provide the first direct evidence for EGL-4 function in the ASH nociceptive neurons, and show that it acts to negatively regulate G protein-coupled signal transduction and dampen behavioral sensitivity to the bitter tastant quinine.

We found that *egl-4(lof)* animals respond better than wild-type animals to 10 mM quinine and also avoid dilute levels of quinine (1 mM) that wild-type animals do not respond to (Figure 1A). EGL-4 function in the two bilaterally symmetric ASHs was both necessary and sufficient to regulate quinine sensitivity (Figures 1B and 1C). Given the short time period that animals are given to initiate the avoidance response (four seconds), we reasoned that EGL-4's primary function in modulating quinine sensitivity is likely the regulation of signal transduction; nuclear translocation and transcriptional changes are unlikely to occur on this timescale. Indeed, in contrast to the necessary nuclear localization of EGL-4 to mediate long-term odor adaptation in AWC [49], cytoplasmic EGL-4 expression in the ASHs was sufficient to rescue the quinine hypersensitivity of *egl-4(lof)* animals (Figure 3A).

Calcium influx is essential for neuronal function and increased intracellular calcium levels trigger exocytosis of neurotransmitter-containing synaptic vesicles [83]. Consistent with their observed enhanced behavioral sensitivity, upon exposure to quinine *egl-4(lof)* animals exhibited an elevated ASH calcium flux, when compared to wild-type animals (Figures 3B and 3C). Combined, our data suggest that EGL-4 normally dampens ASH signaling. Interestingly, this is in contrast to the role of mouse PKGI in centrally located nociceptors in mice. In these cells, PKGI phosphorylation of IP_3_R(Ser1755) potentiates IP_3_R-mediated calcium release from internal stores [84]–[86]. This promotes stimulus-induced synaptic transmission between nociceptors and spinal-periaqueductal grey projection neurons, ultimately leading to the withdrawal reflex (to applied pressure, intrathecally administered NMDA or thermal stimuli) and development of hyperalgesia [84]–[88]. Thus, PKGI promotes an aversive behavioral response in mice, while EGL-4 dampens behavioral sensitivity to a nociceptive stimulus in *C. elegans*. This difference highlights the diversity of mechanisms by which PKGs can regulate signaling in different cellular contexts.

### EGL-4 Negatively Regulates Quinine Sensitivity through RGS-2 and RGS-3

As the *C. elegans* response to quinine utilizes the Gα subunits ODR-3 and GPA-3 [7], [8], our results suggest a role for EGL-4 in the negative regulation of a G protein-coupled signal transduction pathway. We found that when *C. elegans rgs-2(lof)* and *rgs-3(lof)* animals were assayed 30 minutes after being removed from their bacterial food source, they also displayed a significant hypersensitivity phenotype (Figure 4), and RGS-2 and RGS-3 function in ASH was both necessary and sufficient to regulate quinine sensitivity. When the predicted PKG phosphorylation sites in RGS-2 and RGS-3 were changed to alanines, the mutated constructs failed to rescue quinine hypersensitivity. These findings support previous studies using mammalian smooth muscle, astrocyte and fibroblast cells [39]–[42], wherein PKGs can function as activators of RGS proteins, and provide the first evidence of this mechanism in nociceptive sensory neurons. It is also interesting to speculate that EGL-4 may target RGS proteins to regulate additional physiological processes, such as *C. elegans* egg laying [47], [72], [89]–[92]. We examined the amino acid sequences of all twelve *C. elegans* RGS proteins and identified predicted PKG phosphorylation sites in RGS-1, RGS-6, RGS-7, EGL-10 and EAT-16.

The sensitivity of *C. elegans* to environmental stimuli is dramatically and dynamically regulated by an animal's nutritional status, which influences signaling levels of biogenic amines such as serotonin and dopamine [14], [93]. In particular, the responses of wild-type animals to nociceptive stimuli diminish upon food deprivation [28], [64], [94]–[96]. *rgs-3(lof)* animals are defective in their avoidance of 10 mM quinine when assayed just 10 minutes after removal from food (Figure S1) [28]. This defective response is the result of elevated signaling in the ASHs in the absence of the negative regulator, which ultimately leads to decreased synaptic transmission [28]. We see *rgs-3(lof)* animals responding better than wild-type animals to quinine (10 mM and 1 mM) when they are assayed 30 minutes after removal from food (Figure 4). Similarly, *rgs-2(lof)* animals are defective in response to 10 mM quinine when assayed after only the short (10 minute) period of starvation (Figure S1), but are hypersensitive when assayed after being off food for 30 minutes (Figure 4). Interestingly, it is not until 45 minutes off food that *rgs-2(lof);rgs-3(lof)* double mutant animals reach the level of hypersensitivity seen in the single mutants at 30 minutes off food (Figure S1). It is possible that at the intermediate time off food (30 minutes) there is still “too much signaling” in the absence of both RGS-2 and RGS-3 to allow response to 1 mM quinine, similar to the elevated signaling that blocks the response of *rgs-3(lof)* single mutant animals to 10 mM quinine at 10 minutes off food [28]. The longer (45 minute) period off food may allow the increased signaling due to loss of both RGSs to attenuate over time, bringing it into a range that allows for the sensitized behavioral response. Because the time-course for quinine sensitivity differs between *egl-4(lof)* animals and *rgs-2(lof);rgs-3(lof)* animals, EGL-4 may have additional targets within the ASH sensory neurons. Alternatively, RGS-2 and RGS-3 may retain residual function in *egl-4(lof)* animals, such that loss of EGL-4 function does not affect cellular signaling as adversely as loss of the RGS proteins themselves.

### Modulation of Quinine Sensitivity Upstream of EGL-4

**Figure 6:**
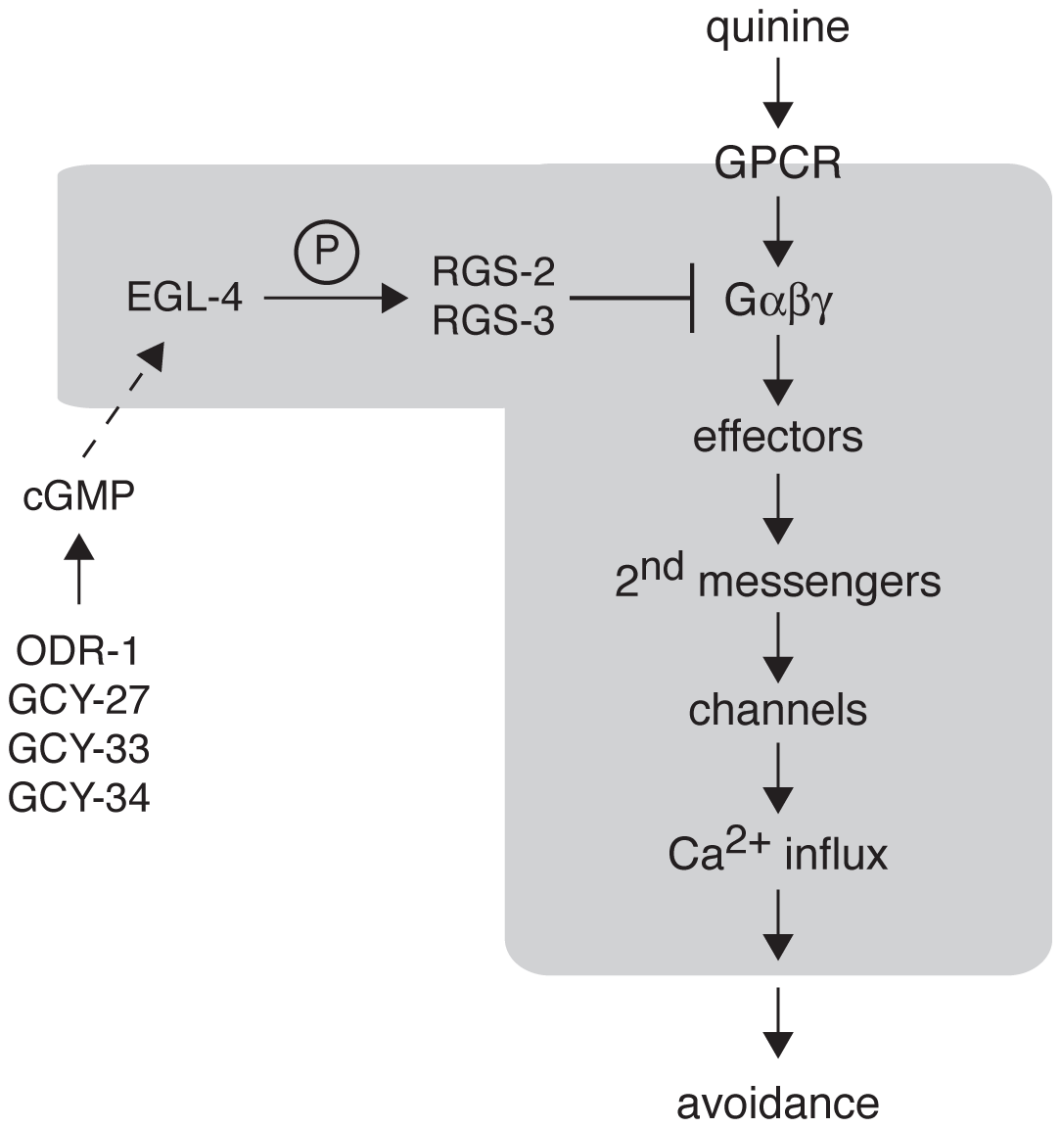
Model for EGL-4 regulation of nociceptive signaling. The cGMP-dependent protein kinase EGL-4 regulates *C. elegans* behavioral sensitivity to the bitter tastants quinine and amodiaquine and the volatile odorant octanol. Wild-type chemosensory signaling is initiated in the ASH sensory neurons when a ligand (such as quinine) binds to a GPCR to activate the associated heterotrimeric G proteins. The activated G proteins (Gα-GTP and Gβγ) interact with downstream effectors to generate second messengers that can activate channels in the plasma membrane, allowing Ca^2+^ influx. Through connections with downstream interneurons and motor neurons, ASH activation is ultimately translated into behavioral avoidance (backward locomotion). Signaling is terminated in part by regulator of G protein signaling (RGS) proteins, which promote the hydrolysis of GTP to GDP by the Gα subunit. EGL-4 phosphorylation of RGS-2 and RGS-3 stimulates their activity. The guanylyl cyclases ODR-1, GCY-27, GCY-33 and GCY-34 may function in alternate neurons to provide the cGMP that is required for EGL-4 function in ASH. In the absence of EGL-4 function, RGS-2 and RGS-3 do not efficiently downregulate Gα signaling, leading to increased Ca^2+^ levels in response to receptor activation. This increased signaling in the ASH sensory neurons leads behavioral hypersensitivity to weak stimuli. The molecular events believed to be happening within the ASHs themselves are included within the grayed area.

PKGs require cGMP for their activation, and the levels of cGMP within a cell are modulated by production by guanlyl cyclases and breakdown by phosphodiesterases. Guanylyl cyclases are widely expressed in mammalian tissues and exist in two forms: soluble and transmembrane [97]. Mammalian soluble GCYs have been well studied in smooth muscle cells, where they are activated by nitric oxide (NO) to produce cGMP [98], [99]. Transmembrane GCYs have been well characterized for their role in natriuresis and phototransduction [100], [101]. While 34 GCYs are encoded by the *C. elegans* genome, the physiological roles of most are unknown. Our analysis revealed that EGL-4 requires cGMP binding in order to negatively regulate quinine sensitivity (Figure 5A), suggesting that a pool of cGMP is available to activate EGL-4 in the ASH nociceptive neurons. Moreover, animals lacking the function of the transmembrane guanylyl cyclases ODR-1 and GCY-27, or the soluble guanylyl cyclases GCY-33 and GCY-34, are hypersensitive in their response to dilute quinine (Figure 5B). However, these GCYs do not appear to function directly in the ASHs (Figure 6), suggesting that other neurons in the circuit may provide the cGMP that regulates ASH function, perhaps via GAP junctions between cGMP-generating neurons and ASH. For example, *gcy-27* is expressed in ASK [59], which forms GAP junctions directly with ASH. Continued studies in *C. elegans* may yield new insights into nociceptive signaling in mammalian systems, where PKGI is known to function in central nociceptors [84]–[86].

## Materials and Methods

### *C. elegans* Culture

Strains were maintained under standard conditions on NGM agar plates seeded with OP50 *E. coli* bacteria [102].

### Strains

Strains used in this study include: N2 Bristol wild-type, MT1074 *egl-4(n479)*, DA521 *egl-4(ad450)*, CX10979 *kyEx2865* (*sra-6p::G-CaMP3;ofm-1p::gfp*), FG414 (*kyEx2865 sra-6p::G-CaMP3;ofm-1p::gfp*), FG417 *egl-4(n479);kyEx2865* (*sra-6p::G-CaMP3;ofm-1p::gfp*), FG413 *egl-4(ad450);kyEx2865* (*sra-6p::G-CaMP3;ofm-1p::gfp*), FG454 *egl-4(n479);udEx208* (*osm-10p::egl-4;myo-3p::mCherry*);*kyEx2865* (*sra-6p::G-CaMP3;ofm-1p::gfp*), LX147 *rgs-1(nr2017)*, LX160 *rgs-2(vs17)*, LX242 *rgs-3(vs19)*, LX533 *rgs-6(vs62)*, FG105 *rgs-10(ok1039)*, FG108 *rgs-10/11(vs109)*, MT8504 *egl-10(md176)*, LX1226 *eat-16(tm761)*, FG329 *egl-4(n479);rgs-2(vs17)*, FG330 *rgs-3(vs19);egl-4(n479)*, FG275 *egl-4(ad450);rgs-2(vs17)*, FG276 *rgs-3(vs19);egl-4(ad450)*, FG269 *rgs-3(vs19);rgs-2(vs17)*, FG376 *rgs-3(vs19);egl-4(n479);rgs-2(vs17)*, VC2796 *gcy-3(gk1154)*, RB1010 *gcy-5(ok930)*, IK800 *gcy-8(oy44)*, VC2675 *gcy-15(gk1102)*, VC2450 *gcy-17(gk1155)*, VC2321 *gcy-18(ok3047);nT1[q1351]*, RB1909 *gcy-19(ok2472)*, RB1935 *gcy-20(ok2538)*, RB924 *gcy-23(ok797)*, VC2375 *gcy-25(gk1187)*, CZ3714 *gcy-31(ok296)*, RB1048 *gcy-32(ok995)*, AX1295 *gcy-35(ok769)*, RB626 *gcy-37(ok384)*, IK597 *gcy-23(nj37);gcy-8(oy44);gcy-18(nj38)*, FG290 *odr-1(n1936)*, FG280 *gcy-27(ok3653)*, FG278 *gcy-33(ok232)*, FG279 *gcy-34(ok2953)*, FG294 *egl-4(ad450);odr-1(n1936),* FG288 *gcy-27(ok3653);egl-4(ad450)*, FG289 *egl-4(ad450);gcy-33(ok232)* and FG285 *egl-4(ad450);gcy-34(ok2953)*.

### Transgenic Strains

Germline transformations were performed as previously described [103]. For *egl-4, rgs-2, rgs-3*, *odr-1*, *gcy-27, gcy-33*, and *gcy-34* rescue experiments, 50 ng/µl of pJM67 *elt-2::gfp* plasmid [104] was used as the co-injection marker, along with 50 ng/µl of the rescuing plasmid. For *rgs-2* and *rgs-3* overexpression, 250 ng/µl of the *rgs* plasmid was co-injected with 50 ng/µl of pJM67 *elt-2::gfp* plasmid [104]. For *egl-4* rescue with G-CaMP3 expression, 5 ng/µl of *myo-3p::mCherry* plasmid (Yamamoto Lab) was used as the co-injection marker, along with 50 ng/µl of the *osm-10p::egl-4* rescuing plasmid. Animals expressing the *egl-4* rescuing array (*udEx208*) were then crossed with *kyEx2865*-expressing (*sra-6p::G-CaMP3;ofm-1p::gfp*) animals, and *egl-4(n479)* animals co-expressing both arrays were isolated. Cell-specific RNAi knock-down experiments were performed as previously described [63]. 25 ng/µl of pJM67 *elt-2::gfp* plasmid [104] was co-injected with 50 ng/µl of each PCR fusion product [63].

### Plasmid Construction

#### pFG1

The ∼900 bp *osm-10* promoter was isolated from CR142 [105] using PstI/BamHI and inserted into these sites of Fire vector pPD49.26 (Fire Lab *C. elegans* Vector Kit, Addgene).

#### pFG50

The ∼2.1 kb *srbc-66* promoter was isolated from *srbc-66::gfp* [62] using HindIII/BamHI and inserted into these sites of Fire vector pPD49.26 (Fire Lab *C. elegans* Vector Kit, Addgene).

#### pFG34 *osm-10p::egl-4*

The *egl-4* cDNA (isoform 2a.1) was PCR amplified from *odr-3::egl-4* [48], incorporating a 5′ XbaI site and a 3′ NcoI, and subcloned into the NheI and NcoI sites of pFG1.

#### pFG99 *srb-6p::egl-4*

The ∼1.3 kb *srb-6* promoter was isolated from pFG10 [106] using SphI/BamHI and inserted into these sites of pFG34 (replacing the *osm-10* promoter).

#### pFG53 *srbc-66p::egl-4*

The *egl-4* cDNA (isoform 2a.1) was PCR amplified from *odr-3::egl-4* [48], incorporating a 5′ XbaI site and a 3′ NcoI, and subcloned into the NheI and NcoI sites of pFG50.

#### pFG97 *osm-10p::rgs-2*

The *rgs-2* cDNA was PCR amplified from pMD6 [72], incorporating a 5′ SalI site and a 3′ NcoI site, and subcloned into these sites of pFG1.

#### pHA446 *osm-10p::rgs-3*

cDNA encoding the “A” isoform of RGS-3, including the initiator methionine, was amplified from yk252h2 (kind gift of Y. Kohara), incorporating a 5′ NheI and a 3′ NcoI site. This fragment was inserted into the Fire vector pPD49.26 (Fire Lab *C. elegans* Vector Kit, Addgene) to create pHA443. The ∼900 bp *osm-10* promoter was isolated from CR142 [105] using Pst-1/Xma-1 and inserted into these sites of pHA443.

#### pFG98 *osm-10p::rgs-2(S126A)*

Site-directed mutagenesis (QuikChange, Stratagene) was used to incorporate the S126A change into pMD6 [72]. The *rgs-2(S126A)* cDNA was then PCR amplified, incorporating a 5′ SalI site and a 3′ NcoI site, and subcloned into these sites of pFG1.

#### pFG93 *osm-10p::rgs-3(S154A)*

Site-directed mutagenesis (QuikChange, Stratagene) was used to incorporate the S154A change into pHA443 (A isoform of RGS-3, above). The *rgs-3(S154A)* cDNA was then PCR amplified, incorporating a 5′ NheI site and a 3′ NcoI site, and subcloned into these sites of pFG1.

#### pFG80 *osm-10p::gfp::egl-4*

SphI/NgoMIV digest was used to remove 2496 bp of the 2678 bp *odr-3* promoter from *odr-3::gfp::egl-4* [49]. The *osm*-10 promoter was isolated from pFG1 using SphI/XmaI and inserted into these sites upstream of *gfp::egl-4*.

#### pFG111 *osm-10p::gfp::egl-4(ΔNLS)*

SphI/NgoMIV digest was used to remove 2496 bp of the 2678 bp *odr-3* promoter from *odr-3::gfp::egl-4(ΔNLS)* [49]. The *osm-10* promoter was isolated from pFG1 using SphI/XmaI and inserted into these sites upstream of *gfp::egl-4(ΔNLS)*.

#### pFG82 *osm-10p::NLS::gfp::egl-4*

SphI/NgoMIV digest was used to remove 2496 bp of the 2678 bp *odr*-3 promoter from *odr-3::NLS::gfp::egl-4* [49]. The *osm-10* promoter was isolated from pFG1 using SphI/XmaI and inserted into these sites upstream of *NLS::gfp::egl-4*.

#### pFG113 *osm-10p::gfp::egl-4(T276A)*

SphI/NgoMIV digest was used to remove 2496 bp of the 2678 bp *odr-3* promoter from *odr-3::gfp::egl-4(T276A)* [49]. The *osm-10* promoter was isolated from pFG1 using SphI/XmaI and inserted into these sites upstream of *gfp::egl-4(T276A)*.

#### pFG133 *osm-10p::gcy-33*

cDNA encoding the “A” isoform of GCY-33, including the initiator methionine, was isolated from *gcy-31p::gcy-33* [107] using NheI/Asp718I and subcloned into these sites of pFG1.

#### pFG134 *srb-6p::gcy-33*

cDNA encoding the “A” isoform of GCY-33, including the initiator methionine, was isolated from *gcy-31p::gcy-33* [107] using NheI/Asp718I and subcloned into these sites of pFG10 [106].

#### pFG138 *osm-10p::gcy-34*

Genomic *gcy-34* sequence was PCR amplified from N2 lysate, incorporating a 5′ NheI site and a 3′ Asp718I site, and subcloned into these sites of pFG1.

#### pFG139 *srb-6p::gcy-34*

The *gcy-34* genomic sequence was isolated from pFG138 using NheI/Asp718I and subcloned into these sites of pFG10 [106].

#### KC3 *odr-1p::odr-1b::gfp*

R01E6.1SC [108] contains the *odr-1* genomic region from 2.6 kb upstream of the start site for *odr-1b* to 79 bp of the last exon (22nd) of *odr-1b*. This construct is missing the last 46 nucleotides of the 22nd exon of *odr-1b* and the 3′ UTR, as predicted by PACdb ID: R01E6.3_216. We added back the last 46 nt and all of the 3′ UTR (449 nt) by first amplifying the 3′ end of *odr-1b* and all of the 3′ UTR from N2 genomic DNA, adding a KpnI site on the 3′ end of the amplicon. This was Topo cloned and cut from the Topo cloning vector with KpnI. This KpnI fragment was inserted into the Kpn1 site of R01E6.1SC [108] to yield KC1. In order to tag ODR-1b with GFP, site-directed mutagenesis was used to add adjacent AgeI and NruI sites at the very last codon of *odr-1b* (before stop) so that *gfp* could be inserted in frame. This construct was called KC2. Finally, *odr-1p::odr-1b::gfp* (KC3) was generated by cutting KC2 with AgeI/NruI and inserting *gfp* (Fire vector pPD95.77 cut with EcoRI, then blunted, then cut with AgeI).

#### pFG140 *osm-10p::odr-1*

Genomic sequence encoding ODR-1 was PCR amplified from KC3 (above), incorporating a 5′ NheI site and a 3′ NcoI site, and subcloned into these sites of pFG1.

#### pFG141 *srb-6p::odr-1*

Genomic *odr-1* sequence was isolated from pFG140 using NheI/NcoI and subcloned into these sites of pFG10 [106].

#### pFG142 *osm-10p::gcy-27*

Genomic *gcy-27* sequence was PCR amplified from N2 lysate, incorporating a 5′ NheI site and a 3′ BsrGI site, and subcloned into the NheI/Asp718 sites of pFG1.

#### pFG143 *srb-6p::gcy-27*

The *gcy-27* genomic sequence was isolated from pFG142 using NheI/ApaI and subcloned into these sites of pFG10 [106].

#### pFG144 *srbc-66p::gcy-27*

The *gcy-27* genomic sequence was isolated from pFG142 using NheI/ApaI and subcloned into these sites of pFG50.

All constructs were verified by sequencing when appropriate.

### Calcium Imaging

Neuronal calcium changes were recorded using the GFP-based fluorescent calcium reporter G-CaMP3 [69], [70] and based on described methods [109], [110]. Briefly, a microfluidic device similar to that used by [110] was fabricated by the Stanford Microfluidics Foundry and used to immobilize a *kyEx2865* (*sra-6p::G-CaMP3;ofm-1p::gfp*)-expressing worm for imaging while streams of buffer or quinine (or buffer for controls) under laminar flow were alternatively presented to the nose of the worm. To reduce the influence of neuronal activation by blue light, we pre-exposed the worm to blue light for 5–8 seconds. The worm was imaged one minute later using a 40X air objective on an inverted Axiovert 200 microscope (Zeiss, Oberkochen, Germany). Images were captured with an exposure time of 20 milliseconds every 500 milliseconds with an ORCA-Flash 2.8 camera (Hamamatsu, Shizuoka Pref., Japan) and recorded over 15 seconds with µManager software [111]. The movies were analyzed using ImageJ (Rasband, W.S., ImageJ, US NIH, Bethesda, MD, USA). The mean signal intensities of the region of interest (ROI) and background were determined and the background corrected value taken for further analysis. The percent change in fluorescence intensity based on the average intensity of the first 3 frames (delta F/F_0_) was calculated. The ROI was centered on the cell body of the ASH neuron. After 5 seconds of imaging with M13 buffer (pH 7.4), the quinine stimulus was given for the last 10 seconds of imaging. Quinine was dissolved as a 1 or 10 mM solution in M13 and the pH was adjusted to 7.4 after dissolving. Ten worms for each condition were tested and averaged. A one-tailed unpaired Student's t-Test for each point in all conditions was performed. The maximum change in fluorescence before and after exposure to quinine was determined and used for statistics.

### Behavioral Assays

Well-fed young adults were used for analysis, and all behavioral assays were performed on at least three separate days, in parallel with controls. The number of transgenic animals assayed in each experiment is indicated within the figure legends, and in all cases n≥58 for non-transgenic animals. Response to the soluble tastant quinine was scored as the percentage of animals that initiated backward locomotion within 4 seconds of encountering a quinine drop placed on the agar plate in front of a forward moving animal [6], [7], [65]. Quinine was dissolved in M13, pH 7.4 [112]. For quinine avoidance assays, animals were tested 30 minutes after transfer to NGM plates lacking bacteria (“off food”). Response to octanol was scored as the amount of time it took an animal to initiate backward locomotion when presented with a hair dipped in octanol [3], [61]. For octanol avoidance assays, animals were tested 10–20 minutes after transfer to NGM plates lacking bacteria and assays were stopped at 20 seconds. All data is presented as ± standard error of the mean (SEM). The Student's two-tailed t-Test was used for statistical analysis, except for panels 4A and 5B, in which the one-way Anova with Tukey's Honestly Significant Difference (HSD) statistical analysis was used.

## Supporting Information

Figure S1 Length of starvation alters *C. elegans* sensitivity to quinine. Animals lacking EGL-4, RGS-2, RGS-3 or RGS-2 and RGS-3 function were tested for response to both (A) 10 mM quinine and (B) 1 mM quinine when starved for increasing lengths of time. (A) *rgs-2(lof)* and *rgs-3(lof)* animals responded to 10 mM similarly to wild-type animals when assayed 30 minutes after removal from their bacterial food source (p>0.5 and p>0.1, respectively). *rgs-2(lof);rgs-3(lof)* double mutant animals did not respond similarly to wild-type animals until 60 minutes after removal from food (p>0.5). (B) The behavioral sensitivity of *rgs-2(lof)*, *rgs-3(lof)* and *rgs-2(lof);rgs-3(lof)* animals to dilute quinine increased with longer periods off food. The percentage of animals responding is shown. The combined data of n≥40 animals is shown. Error bars represent the standard error of the mean (SEM). Alleles used: *egl-4(n479)*, *rgs-2(vs17)*, and *rgs-3(vs19)* loss-of-function. WT = the N2 wild-type strain. lof = loss-of-function, min = minutes.
(PDF)
