# Supplementary figures and images for: The C. elegans cGMP-Dependent Protein Kinase EGL-4 Regulates Nociceptive Behavioral Sensitivity
Source: PLoS Genet. 2013 Jul 11;9(7):e1003619. doi: 10.1371/journal.pgen.1003619 (PMC3708839; doi:10.1371/journal.pgen.1003619)

## Supplemental Data

Figure S1

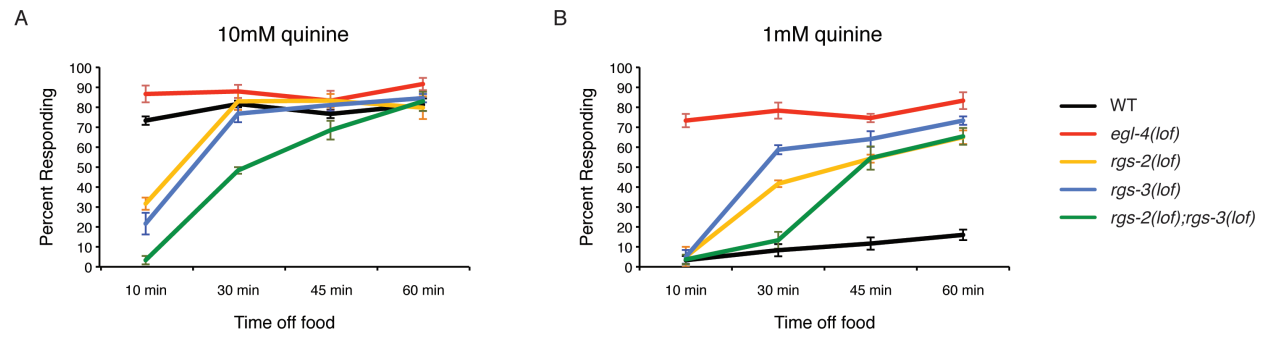

Supplement: Figure S1 — Length of starvation alters C. elegans sensitivity to quinine. Animals lacking EGL-4, RGS-2, RGS-3 or RGS-2 and RGS-3 function were tested for response to both (A) 10 mM quinine and (B) 1 mM quinine when starved for increasing lengths of time. (A) rgs-2(lof) and rgs-3(lof) animals responded to 10 mM similarly to wild-type animals when assayed 30 minutes after removal from their bacterial food source (p>0.5 and p>0.1, respectively). rgs-2(lof);rgs-3(lof) double mutant animals did not respond similarly to wild-type animals until 60 minutes after removal from food (p>0.5). (B) The behavioral sensitivity of rgs-2(lof), rgs-3(lof) and rgs-2(lof);rgs-3(lof) animals to dilute quinine increased with longer periods off food. The percentage of animals responding is shown. The combined data of n≥40 animals is shown. Error bars represent the standard error of the mean (SEM). Alleles used: egl-4(n479), rgs-2(vs17), and rgs-3(vs19) loss-of-function. WT = the N2 wild-type strain. lof = loss-of-function, min = minutes. (PDF) [file pgen.1003619.s001.pdf]
